# Sustained attention operates via dissociable neural mechanisms across different eccentric locations

**DOI:** 10.1038/s41598-024-61171-7

**Published:** 2024-05-16

**Authors:** Tanagrit Phangwiwat, Phond Phunchongharn, Yodchanan Wongsawat, Itthi Chatnuntawech, Sisi Wang, Chaipat Chunharas, Thomas C. Sprague, Geoffrey F. Woodman, Sirawaj Itthipuripat

**Affiliations:** 1 Neuroscience Center for Research and Innovation (NX), Learning Institute, King Mongkut’s University of Technology Thonburi (KMUTT), Bangkok, 10140 Thailand; 2 Big Data Experience Center (BX), King Mongkut’s University of Technology Thonburi (KMUTT), Bangkok, 10600 Thailand; 3 Department of Computer Engineering, King Mongkut’s University of Technology Thonburi (KMUTT), Bangkok, 10140 Thailand; 4 Department of Biomedical Engineering, Faculty of Engineering, Mahidol University, Nakhon Pathom, 73170 Thailand; 5 National Nanotechnology Center, National Science and Technology Development Agency, Pathum Thani, 12120 Thailand; 6 Department of Experimental and Applied Psychology, Vrije Universiteit Amsterdam, Amsterdam, The Netherlands; 7 Department of Psychology, Vanderbilt University, Nashville, TN 37235 USA; 8 Cognitive Clinical and Computational Neuroscience Center of Excellence, Department of Internal Medicine, Faculty of Medicine, Chulalongkorn University, Bangkok, 10330 Thailand; 9 Chula Neuroscience Center, King Chulalongkorn Memorial Hospital, Thai Red Cross Society, Bangkok, 10330 Thailand; 10 Department of Psychological and Brain Sciences, University of California Santa Barbara, Santa Barbara, CA 93106 USA

**Keywords:** Attention, Eccentricity, Foveal vision, Peripheral vision, EEG, SSVEP, Visual cortex, Frontoparietal cortex, Neuroscience, Psychology

## Abstract

In primates, foveal and peripheral vision have distinct neural architectures and functions. However, it has been debated if selective attention operates via the same or different neural mechanisms across eccentricities. We tested these alternative accounts by examining the effects of selective attention on the steady-state visually evoked potential (SSVEP) and the fronto-parietal signal measured via EEG from human subjects performing a sustained visuospatial attention task. With a negligible level of eye movements, both SSVEP and SND exhibited the heterogeneous patterns of attentional modulations across eccentricities. Specifically, the attentional modulations of these signals peaked at the parafoveal locations and such modulations wore off as visual stimuli appeared closer to the fovea or further away towards the periphery. However, with a relatively higher level of eye movements, the heterogeneous patterns of attentional modulations of these neural signals were less robust. These data demonstrate that the top-down influence of covert visuospatial attention on early sensory processing in human cortex depends on eccentricity and the level of saccadic responses. Taken together, the results suggest that sustained visuospatial attention operates differently across different eccentric locations, providing new understanding of how attention augments sensory representations regardless of where the attended stimulus appears.

## Introduction

The primate visual system comprises multiple cortical structures that are highly organized and interconnected^1–6^. Neurons within these cortical structures form retinotopic maps, enabling simultaneous encoding and integration of visual inputs across the visual field^7–10^. To efficiently process fine-grained visual information, the visual system has developed a dual neural architecture, with higher temporal sensitivity in the periphery and higher spatial sensitivity in the fovea^11–13^. This functional division allows humans to engage in tasks such as reading road signs while simultaneously monitoring the surrounding environment for vehicles and pedestrians.

To navigate through the environment, the visual system also requires selective attention to prioritize sensory inputs that are most relevant to behavioral goals. Although the differences in neuroanatomy between central and peripheral vision have been well-documented, it is still debated whether attention operates through the same or different neural mechanisms across eccentricities. A study of attention in nonhuman primates proposed that attention operated through different neural mechanisms across the foveal and peripheral locations^14^). Specifically, they found that attention increased the size of receptive fields (RFs) and the attentional gain of neurons in the primary visual cortex, whose RFs overlapped with attention and visual stimuli located at peripheral locations^14^. In contrast, reduced RF size and negligible attentional gain were observed near the fovea^14^. These findings suggest that the effects of attention on visuocortical processing are not uniform across eccentricities, and that foveal and peripheral attention may involve distinct cortical mechanisms, necessitating varying levels of perceptual demands and spatial integration of sensory information.

However, a later human EEG study found contradictory results, where a significant gain amplification of the early sensory response measured at the parafoveal locations was observed^15^. They used this evidence to argue that foveal and peripheral attention operated via similar neural gain amplification mechanisms^15^. However, this EEG study did not systematically examine the degree of attentional modulations as a function of eccentricity, calling into question whether the early sensory evoked responses undergo the same or different levels of attentional modulations across visual space.

Further complicating matters, fMRI studies have reported inconsistent results^10,16^. In one study, the degree of attentional modulations of hemodynamic responses measured in early visual areas was much greater at the fovea compared to the more peripheral locations^16^. In contrast, in another fMRI study, opposing results were observed^10^. Specifically, in this study, the degree of attentional modulations on the amplitude of spatially selective sensory representations, based on the pattern of fMRI activity measured in early visual areas, did not change as a function of eccentricity^10^. These discrepant fMRI results could be due to eye movements since they were not monitored in these studies. This is likely because past studies have shown that eye movement control is supported by the prefrontal network and can cause changes in neural activity in up-stream visual areas^17–22^.

It is important to note that attentional modulations in fMRI signals in the early visual cortex reflect top-down synaptic inputs from the higher-order attentional control regions to early visual areas and do not track the interaction between attention and sensory evoked responses or spiking outputs from the early visual areas per se^23–27^. Consistent with this idea, previous fMRI studies have shown that attention enhances fMRI activity in early visual areas even without the presence of a visual stimulus, and the degree of attentional modulations of fMRI activity is independent of stimulus intensity^27–33^. Thus, in addition to the contradictory findings in the fMRI literature, it remains unanswered whether the same or different neural mechanisms underlie the effects of selective attention on early sensory processes that occur at different eccentric locations.

Here, we aimed to distinguish between the competing accounts of the operation of attention mechanisms across different eccentric locations in the visual field. To do so, we monitored attention-induced changes in two EEG markers commonly used to index visual information processing, while human subjects attended to visual stimuli presented at different eccentric locations (i.e., attend-stimulus) or attended at the central fixation (i.e., attend-fixation). These EEG markers were the steady-state visually evoked potential (SSVEP) and the sustained negative deflection (SND) in the event-related potential (ERP), thought to track early sensory and top-down attentional control processes, respectively. The attentional modulations of the SSVEP signals are thought to index the attentional gain enhancement of the synchronous stimulus-evoked responses generated from the early visual areas^34,35^. On the other hand, the SND, which is the slow negative-going ERP wave, has been proposed to measure the top-down control signals from the fronto-parietal cortex onto the upstream visual areas^27,36–39^. To control for task difficulty, we equated accuracy levels across different attention tasks (i.e., attend-stimulus vs. attend-fixation) and different eccentricity levels. We also monitored eye movements via electrooculography (EOG) and sorted trials based on the level of eye movements in order to examine the effect of eye movements on the patterns of attentional modulations of the SSVEP and SND data.

With a minimal level of eye movements, we found that the degree of attentional modulations of the SSVEP and SND responses were heterogeneous across eccentric locations. Specifically, the attentional modulations of these neural signals peaked at the parafoveal locations but were reduced as visual stimuli got closer to the fovea or appeared further away from the parafoveal locations towards the peripheral direction. That said, in trials with a relatively higher level of eye movements, the heterogeneous patterns of attentional modulations of the SSVEP and SND data were less robust or even became homogeneous across eccentricities. Together, our findings suggest that sustained visuospatial attention operates differently across different eccentric locations and the contradictory findings in past literature could be due to confounds from differences in the level of eye movement artifacts across studies.

## Results

**Figure 1:**
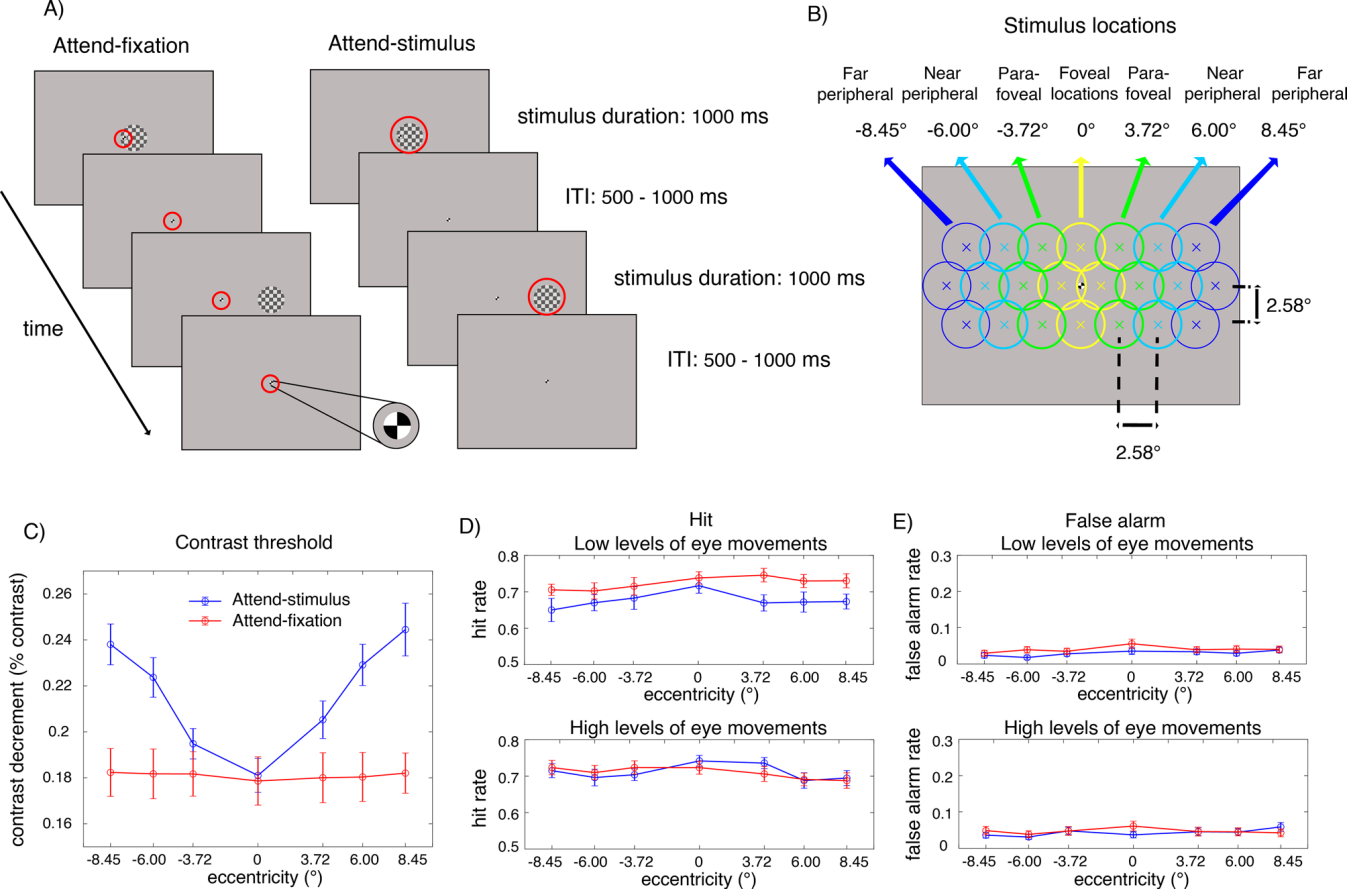
Behavioral tasks and results. (**A**) The sustained spatial attention tasks. (Left in **A**) In the attend-fixation task, subjects were instructed to fixate at a small 100%-contrast checkerboard stimulus located at the center of the screen to detect a rare contrast decrement (20% target trials), while ignoring a large 50%-contrast checkerboard stimulus presented at 1 of 22 possible locations: 4 foveal, 6 parafoveal, 6 near peripheral, and 6 far peripheral locations, marked by yellow, green, cyan, and blue circles in (**B**), respectively. (Right in **A**) In the attend-stimulus task, subjects covertly attended to the large 50%-contrast checkerboard stimulus that could appear at any of the 22 locations to detect a rare contrast dimming at the stimulus location (20% target trials). Red circles (not physically presented) indicate areas on the screen where subjects were instructed to pay attention to (**C**) contrast thresholds plotted as a function of eccentricity in the attend-stimulus and attend-fixation conditions. (**D**,**E**) Hit and false alarm rates plotted as a function of eccentricity in trials with low and high levels of eye movements, respectively. The error bars in (**C**–**E**) represent the within-subject standard errors of the mean (SEMs).

In the present study, we recorded behavioral and EEG responses from human subjects performing a covert visuospatial attention task, where they either attended to a 50%-contrast flickering checkerboard stimulus presented at 1 of 22 possible locations along an elongated hexagonal grid spanning ~ 2.58° × 9.03° visual angle across the entire computer screen or attended to a 100%-contrast central fixation (see detail in Fig. 1A,B and the “Materials and methods” section). In the attend-stimulus blocks, they were instructed to fixate at the central fixation, covertly shift their attention to the stimulus location without producing eye movements and sustain their attention at the stimulus location in order to detect a contrast dimming that could occur in 20% of the entire trials. To monitor SSVEPs, the 50%-contrast checkerboard stimulus was flickered at 18.75 Hz for 1000 ms. In order to ensure that subjects sustained their attention throughout the entire stimulus duration, the contrast dimming event could appear any time from 150–650 ms after the stimulus onset for 350 ms. Intertrial intervals (ITI) were also jittered from 500–1000 ms so that subjects could not predict stimulus onset. The stimulus presentation in the attend-fixation blocks was identical to that in the attend-stimulus blocks except that the contrast dimming appeared at the central fixation and subjects were instructed to attend to the fixation throughout the entire block and ignore the presence of the 50%-contrast checkerboard stimulus that could appeared at 1 of the 22 possible locations. Here, the subjects’ task was to detect contrast dimming at the central fixation that could in 20% of the entire trials.

In order to control the level of task difficulty across attention conditions and eccentric locations, we adjusted the degrees of contrast decrement (i.e., contrast thresholds) on a block-by-block basis to maintain hit rate at around 0.7. To do this, we grouped the 22 stimulus locations into 7 sets of eccentricities and adjusted the contrast thresholds separately for the visual stimuli that fell into these individual groups: − 8.45°, − 6°, − 3.72°, 0°, + 3.72°, + 6° and + 8.45° visual angle, where 0° is the fovea and – and + values indicate the averaged eccentricity relative to the left and right of the fovea (Fig. 1B). Here we termed stimuli that fell into 0°, ± 3.72°, ± 6° and ± 8.45° eccentricities foveal, parafoveal, near peripheral, and far peripheral stimulations, respectively.

### Behavioral results

As illustrated in Fig. 1C, in the attend-fixation condition, we found that the level of contrast thresholds measured at the fixation did not change significantly as the eccentricity of the competing visual stimulus increased (F(6, 162) = 0.13, p = 0.992). However, in the attend-stimulus condition, the contrast threshold significantly increased as a function of eccentricity (F(6,162) = 26.11, p < 0.001). This result is consistent with the increased receptive field (RF) sizes and decreased cortical magnification factors of neurons in the early visual areas as a function of eccentricity^7,9, 40–47^.

The difficulty levels of contrast detection were well maintained at ~ 0.7 hit rate across the different attention conditions and eccentricities (mean hit rate = ± SD = 0.70 ± 0.016) (Fig. 1D). There was a slight but significant main effect of eccentricity on hit rate (range = 0.69–0.73 across all locations; F(6, 162) = 2.80, p = 0.013). Post-hoc paired *t*-tests show that hit rate in the foveal locations were significantly higher than all parafoveal and peripheral locations (t(27)’s = 2.19–3.22, p’s = 0.003–0.037, two-tailed due to the known direction of the attention effect, Holm–Bonferroni corrected with the threshold of 0.05). However, there was neither a significant main effect of attention F(1, 27) = 3.21, p = 0.085 nor a significant interaction between attention and eccentricity on hit rate F(6, 162) = 0.35, p = 0.912.

**Figure 2:**
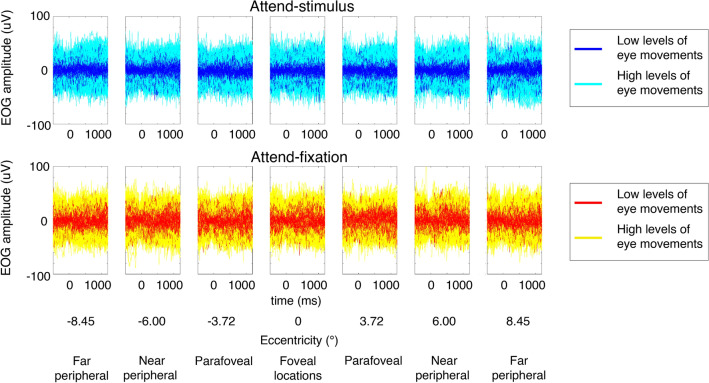
The trial-by-trial EOG signals plotted across all subjects. The EOG data were divided into trials with low vs. high levels of eye movements using the median split method.

To examine the influence of eye movements, we sorted trials based on the level of EOG signals (see Fig. 2 and “Materials and methods”). As illustrated in Fig. 1D, we found that there was a significant interaction between the level of eye movements and attention F(1, 27) = 4.32, p = 0.047. This is driven by a significant main effect of attention in trials with low levels of eye movements (F(1, 27) = 4.77, p = 0.038) but no significant main effect of attention in trials with high levels of eye movements (F(1, 27) = 0.02, p = 0.899). Post-hoc *t*-tests revealed that in trials with lower levels of eye movements, the attention effects on hit rate were significant at the parafoveal locations (t(27) = 2.74, p = 0.005, one-tailed due to the known direction of the attention effect, Holm–Bonferroni corrected with the threshold of 0.0125) and nearly significant at the peripheral locations (t(27)’s = 1.67–1.71, p’s = 0.049–0.053, one-tailed) but not significant at the foveal locations (t(27) = 0.79, p = 0.218, one-tailed). On the other hand, there was no significant attention effect on hit rate at any location for trials with high levels of eye movements (t(27)’s = 0.38–0.87, p’s = 0.197–0.354, one-tailed), suggesting that small saccadic responses were necessary for subjects to maintain similar levels of hit rate across eccentricities in the attended-stimulus condition due reduced contrast sensitivity to visual stimuli in the parafoveal and peripheral locations (see the contrast thresholds results where higher degrees of contrast changes were required for subjects to perform the contrast detection task in Fig. 1C).

As illustrated in Fig. 1D, the false alarm rate in general was relatively low (mean false alarm rate ± SD = 0.036 ± 0.008). There was a slight but significant eccentricity effect of false alarm rate (range = 0.024–0.048 across all locations; F(6, 162) = 5.37, p < 0.001). That said, there was no significant main effect of attention (F(1, 27) = 0.51, p = 0.481) and no significant interactions between attention, eccentricity, and the level of eye movements (F’s = 1.78–1.97, p’s = 0.073–0.194).

### Steady-state visually evoked potential (SSVEP) results

To monitor the effect of sustained visual attention on the stimulus-evoked neural activity in the early visual cortex, we measure SSVEPs, which were the phase-locked visually evoked EEG responses that oscillated at the same frequency as of the frequency of the flickering visual stimulus (i.e., 18.75 Hz). We used the SSVEP technique here because it is thought to capture the population-level visually evoked responses generated from the early visual areas^27,34, 38, 48–57^. Moreover, many past studies have consistently shown that SSVEP is a sensitive measure for neural gain in many variants of sustained visual attention tasks^27,38, 48–58^.

**Figure 3:**
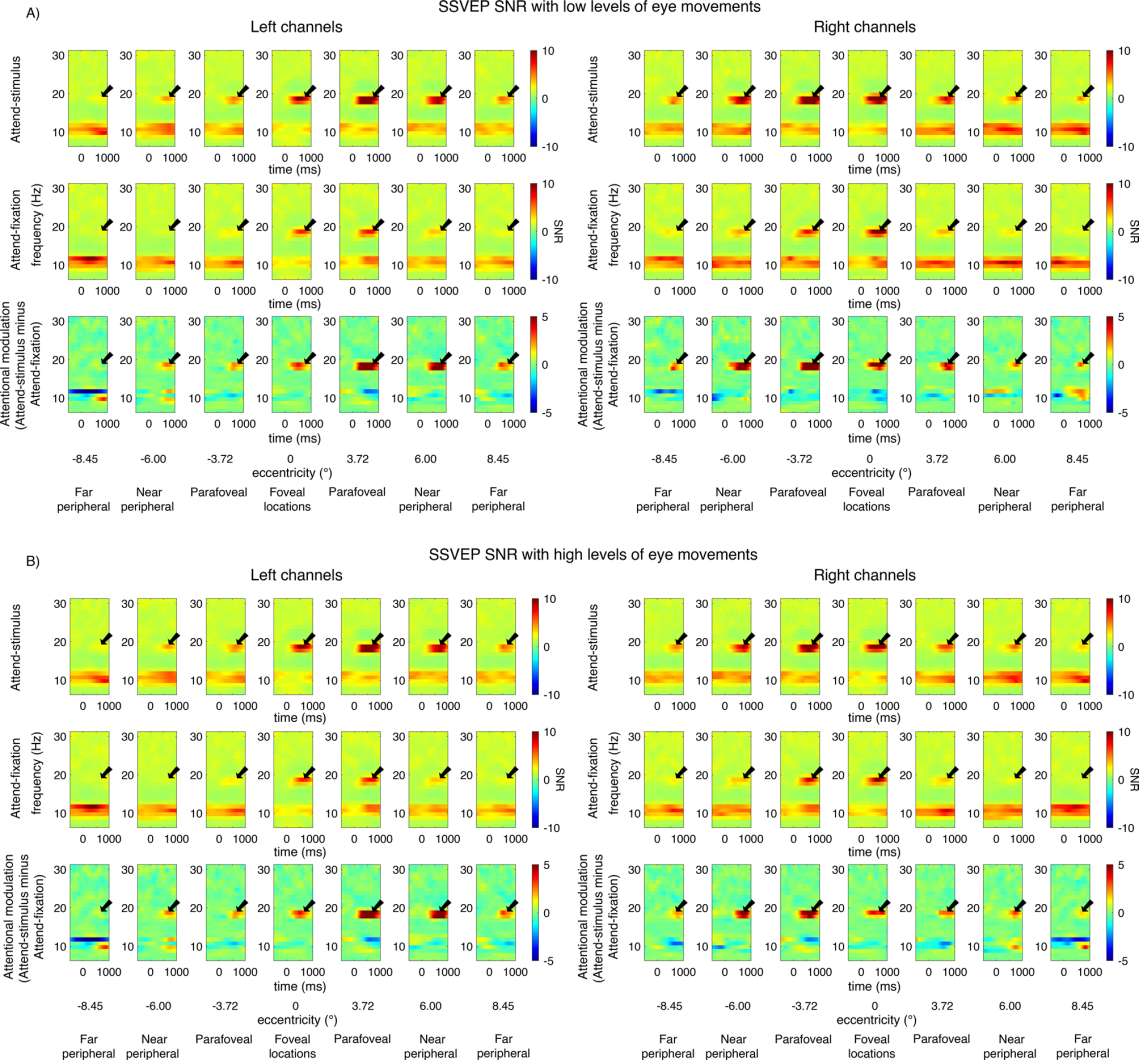
The event-related time frequency plots of the steady-state visually evoked potential (SSVEP) data across different attention conditions and eccentric locations from the left and right posterior occipital electrodes in trials with low vs. high levels of eye movements based on the EOG data in Fig. 2. The SSVEP signals peaked at the stimulus flicker frequency (i.e., 18.75 Hz) as indicated by the black arrows.

**Figure 4:**
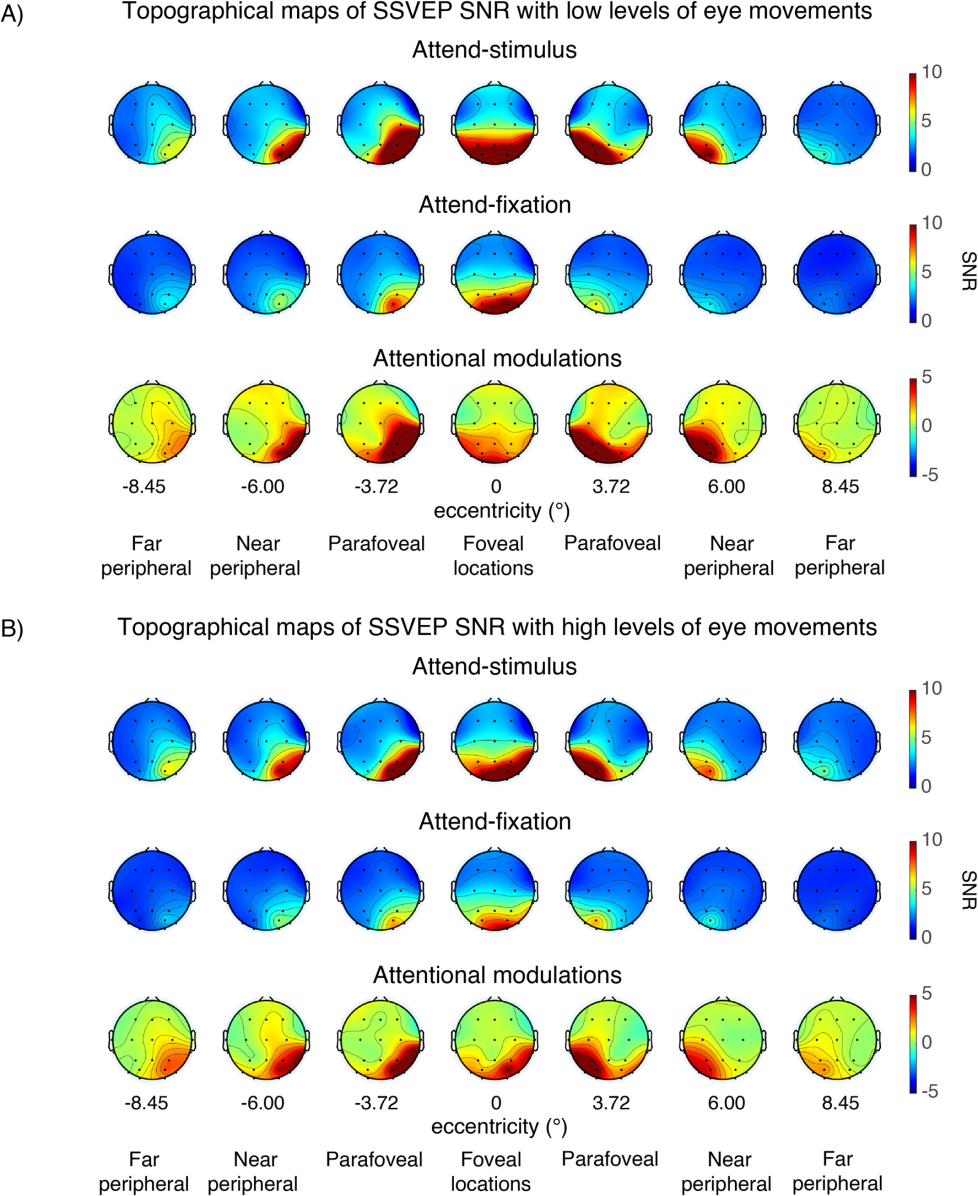
The topographical maps of the steady-state visually evoked potential (SSVEP) data (averaged from 300–1000 ms post-stimulus onset) across different attention conditions and eccentricities in trials with low vs. high levels of eye movements based on the EOG data in Fig. 2.

**Figure 5:**
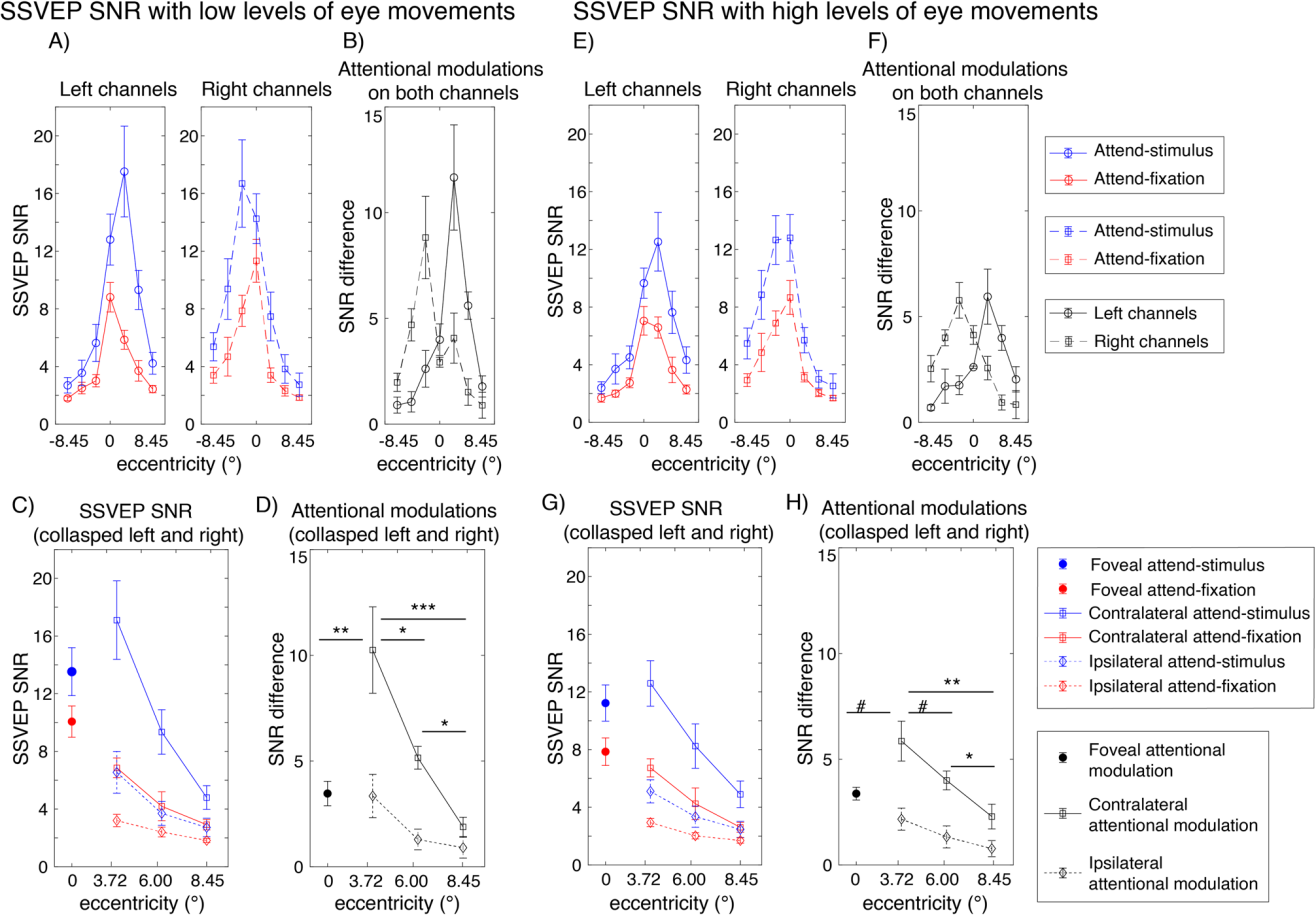
Attentional modulations of the SSVEP signals across eccentricity in trials with low and high levels of eye movements (**A**–**D** and **E**–**H**, respectively). (**A**) SSVEP SNR in the attend-stimulus and attend-fixation conditions measured from the left and right posterior occipital electrodes plotted as a function of eccentricity in trials with low levels of eye movements. (**B**) The difference plot showing the degrees of attention modulations (attend-stimulus minus attend-fixation) from both sets of electrodes plotted in (**A**). (**C**) Same as (**A**) but the data were collapsed across the left and right electrodes. (**D**) Same as (**B**) but the data were collapsed across the left and right electrodes. (**E**–**H**) Similar to (**A**–**D**) but the data were obtained from trials with high levels of eye movements. The error bars in all sub-figures represent the within-subject standard errors of the mean (SEMs). #, *, **, and *** show marginal and significant differences in attentional modulations across eccentric locations with p’s < 0.1, 0.05, 0.01 and < 0.001, respectively (two-tailed).

Consistent with previous reports^27,38, 48–58^, we observed robust SSVEP signals peaking at the flicker frequency of 18.75 Hz and the spectral power at the SSVEP frequency peaked at the posterior occipital electrodes (Figs. 3, 4). For the visual stimuli appearing within the fovea, the SSVEP signals were distributed bilaterally at the posterior occipital sites. However, as the stimuli were presented at the parafoveal and peripheral locations, the SSVEP signals shifted towards the contralateral compared to the ipsilateral electrodes, resulting in a significant interaction between eccentricity and channel location (i.e., left and right posterior occipital electrodes) (F(6,162) = 23.97, p < 0.001) (see Fig. 5). Overall, the mean SNR of the bilateral SSVEP signals elicited by the foveal stimuli were comparable to those of the contralateral SSVEP signals elicited by the parafoveal stimuli (t(27) = − 0.39, p = 0.698, two-tailed, not passing the Holm–Bonferroni-corrected threshold of 0.05). However, the SNR of the contralateral SSVEP signals reduced significantly as the visual stimuli appeared in the eccentric locations further in the periphery, resulting in a significant main effect of eccentricity on the SSVEP SNR (F(6,162) = 34.49, p < 0.001). Post-hoc paired *t*-tests showed that the SSVEP SNR for the foveal and parafoveal locations were significantly higher than those for the near and far peripheral locations (t(27)’s = 4.28–7.06, p’s < 0.001, two-tailed). In addition, the SSVEP SNR was significantly higher for the near compared to the far peripheral locations (t(27) = 4.30, p < 0.001, two-tailed, all tests passed the Holm–Bonferroni-corrected threshold of 0.025). Taken together, these results were consistent with the fact that foveal vision has higher cortical magnification than peripheral vision^7,9, 40, 43, 44, 47^.

As expected, attention significantly increased the SNR of the SSVEP signals (F(1,27) = 18.17, p < 0.001). Importantly, there were significant interactions between attention and eccentricity (F(6,162) = 7.23, p < 0.001) as well as between these factors and channel locations (F(6,162) = 8.62, p < 0.001), showing that the pattern of attentional modulations of SSVEP data was heterogeneous across eccentricities (Fig. 5A–D). As illustrated in Fig. 5D,H, these interactions could be described by two characteristics of attentional gain patterns of the SSVEP results across eccentricities: (i) the reduction of attentional modulations of the SSVEP signals at the foveal compared to the parafoveal locations (t(27) = − 2.85, p = 0.008, two-tailed) and (ii) the reduction of the attentional modulations at the more peripheral direction compared to the parafoveal locations that occurred in a graded fashion (t(27) = 3.59, p = 0.001 for far peripheral vs. parafoveal location; t(27) = 2.90, p = 0.007 for far vs. near peripheral locations, all tests were two-tailed and passed the Holm–Bonferroni-corrected threshold of 0.025).

The SNR of the SSVEP signals also depends on the level of eye movements as the SSVEP SNR was significantly higher in trials with low compared to high levels of eye movements (F(1,27) = 21.6, p < 0.001) (compare Fig. 5A and C to Fig. 5E and G). Importantly, the heterogeneous pattern of attentional modulations across eccentricities reported above was relatively more robust in trials with low levels of eye movements compared to those with high levels of eye movements, resulting in a significant three-way interaction between attention, eccentricity, and the level of eye movements (F(6,162) = 2.44, p = 0.028) (compare Fig. 5B and D to 5F and H). As illustrated in Fig. 5D, in trials with low levels of eye movements, there was a significant reduction in the SSVEP SNR at the foveal compared to the parafoveal locations (t(27) = − 2.80, p = 0.009, two-tailed). Also, the SSVEP SNR significantly reduced as visual stimuli appeared in the locations peripheral to the parafoveal locations (near peripheral vs. parafoveal location: t(27) = 2.60, p = 0.015; far peripheral vs. parafoveal location: t(27) = 3.97, p < 0.001; far vs. near peripheral locations: t(27) = 3.47, p = 0.002, all tests were two-tailed and passed the Holm–Bonferroni-corrected threshold of 0.05). For trials with high levels of eye movements, there was only a marginal difference between the SNR level of the SSVEP signals across the foveal and parafoveal locations (t(27)’s = − 2.04, p = 0.052, two-tailed) (Fig. 5H). The reduction of the SSVEP SNR towards the periphery was significant but relatively less robust as there were significant SNR differences between parafoveal and far peripheral locations (t(27) = 3.55, p = 0.001, two-tailed) and between the near and far peripheral locations (t(27) = 2.38, p = 0.025, two-tailed) but no difference between the parafoveal and near peripheral locations (t(27) = 1.77, p = 0.088, all tests were two-tailed with the Holm–Bonferroni-corrected threshold of 0.025). Taken together, the SSVEP results suggested that the attention effects of early sensory responses as measured by SSVEPs were heterogeneous across eccentricities. Moreover, eye movements affected the SNR level of the SSVEP signals and the robustness of the heterogeneous pattern of attentional modulations of the SSVEP data measured as a function eccentricity.

**Figure 6:**
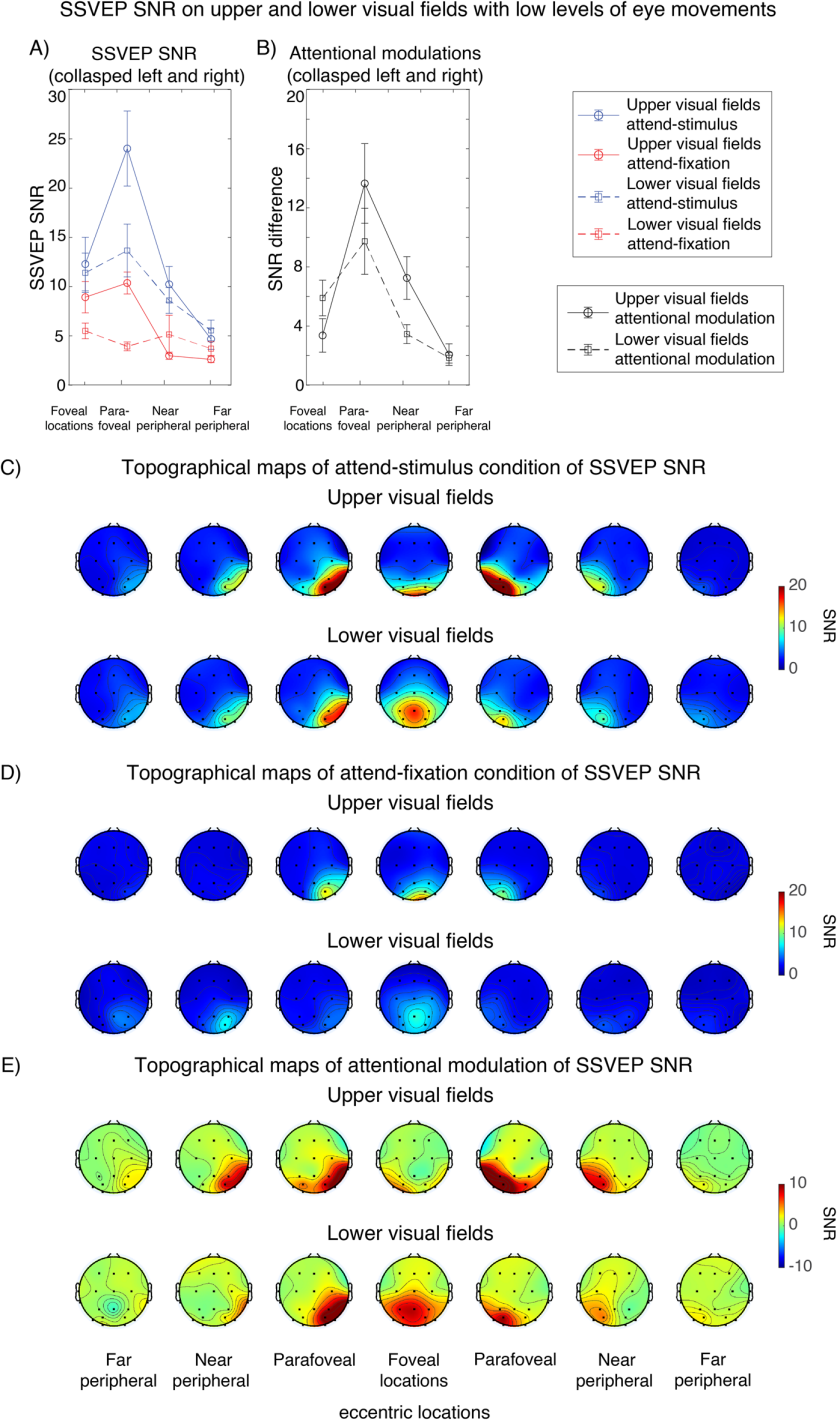
Attentional modulations of the SSVEP signals across eccentricity analysed separately for visual stimuli on the upper and lower visual fields (VFs) in trials with low levels of eye movements. (**A**) SSVEP SNR in the attend-stimulus and attend-fixation conditions collapsed across the left and right posterior occipital electrodes plotted as a function of eccentricity. (**B**) The difference plot showing the degrees of attention modulations (attend-stimulus minus attend-fixation) from (**A**). (**C**–**E**) The topographical maps of the SSVEP data (averaged from 300–1000 ms post-stimulus onset) across different attention conditions (attend-stimulus, attend-fixation, and attention modulation respectively) and eccentricities plotted separately for the upper and lower VF stimulation.

In addition, we conducted an auxiliary analysis where we examined SSVEP modulations separately for visual stimuli in the upper and lower visual fields (VFs) to prevent SSVEP signals associated with these two vertical portions from canceling out. We found that the modulatory patterns of SSVEP results in the upper and lower VFs are qualitatively similar; i.e., we observed heterogeneous patterns of attentional modulations of SSVEP SNR across eccentricity (Fig. 6). Overall, repeated-measures ANOVAs revealed significant main effects of attention (F(1,27) = 21.83, p < 0.001), eccentricity (F(3,81) = 20.56, p < 0.001), and vertical position (upper vs. lower VFs: F(1,27) = 9.31, p = 0.005). Specifically, we found that the SSVEP SNR was higher for upper VF stimulation compared to lower VF stimulation. Importantly, we found that attention significantly interacted with eccentricity (F(3,81) = 8.84, p < 0.001), but it did not interact with vertical position (F(1,27) = 0.81, p = 3.757). Other sets of repeated-measures ANOVAs also showed significant interactions between attention and eccentricity for the data obtained separately from the upper and lower VFs (F(3,81)’s = 8.17 and 5.00, respectively, p’s < 0.001), confirming that they both exhibited the similar heterogeneous patterns of attentional modulations. Thus, the SSVEP results from the lower and upper VFs support the main results that the attentional effect on early sensory responses differed across different eccentric locations.

### Sustained negative deflection (SND) results

Next, we examined the slow negative-going wave in the ERP data, termed here as the sustained negative deflection (SND). The negative deflection in the ERP like the SND component has been found to track the locus of spatial attention in behavioral tasks where the stimulus appeared at the peripheral locations^27,36–39^. Specifically, these studies found that attending to the peripheral locations increased the amplitude of the negative deflection (i.e., it becomes more negative) in the contralateral posterior occipital electrodes^27,38, 39^. Importantly, a seminal study combining EEG and fMRI demonstrated that the SND component reflects the top-down attentional biasing signals from the fronto-parietal cortex onto the upstream visual areas^36^.

**Figure 7:**
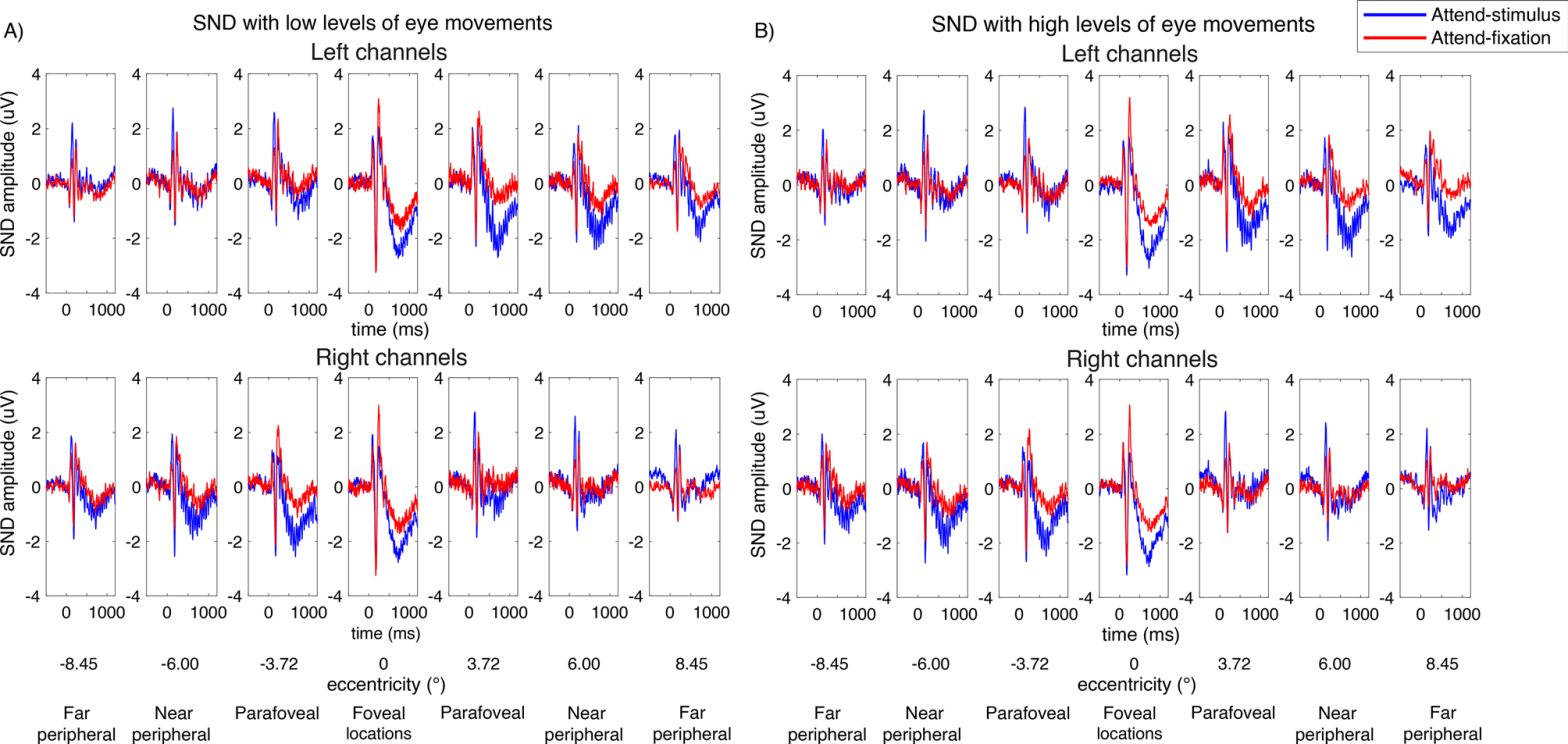
SND traces across different attention conditions and eccentricities from the left and right posterior occipital electrodes in trials with low vs. high levels of eye movements based on the EOG data in Fig. 2. The shading areas represent the within-subject standard errors of the mean (SEMs).

**Figure 8:**
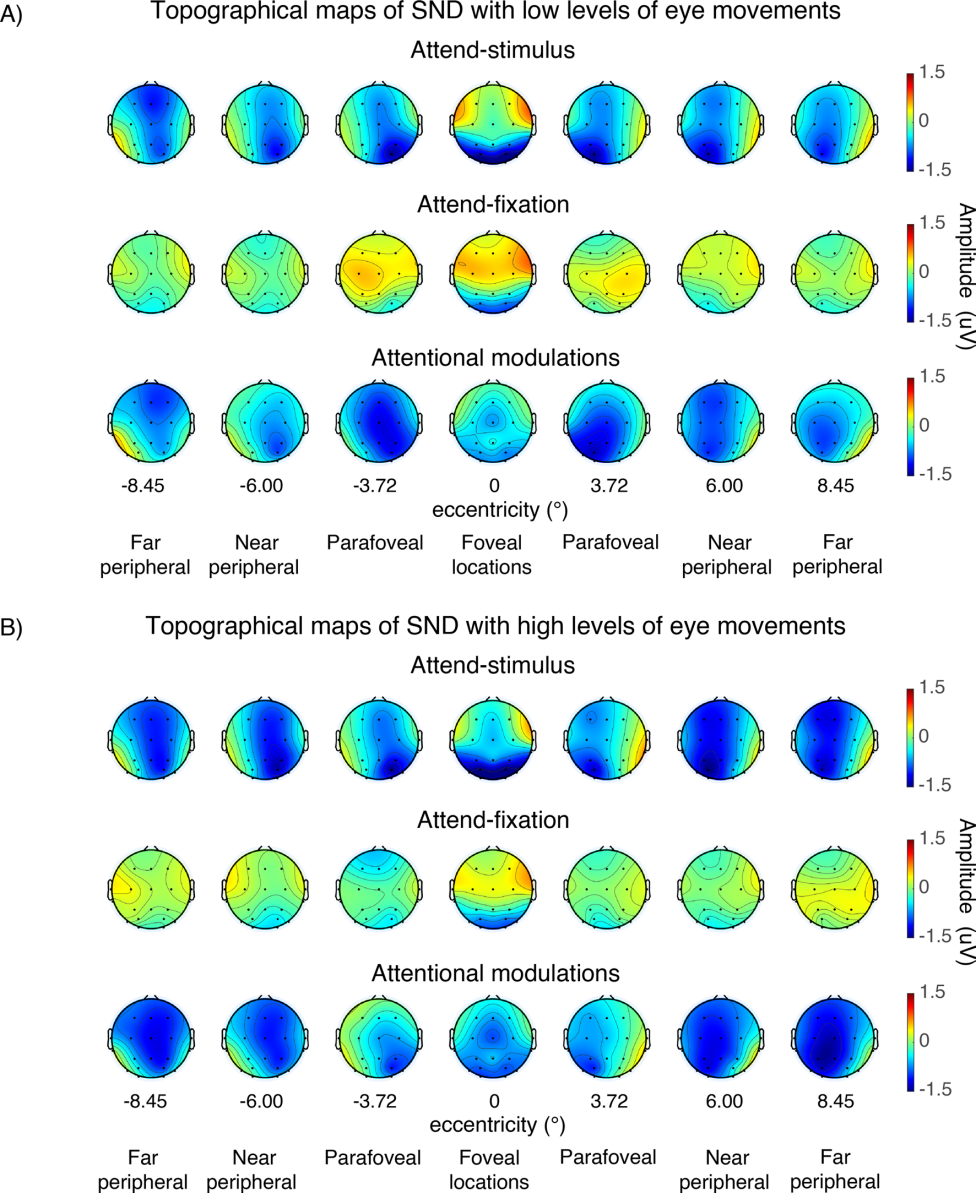
The topographical maps of the SND amplitudes (averaged from 300–1000 ms post-stimulus onset) of the data shown in Fig. 7.

**Figure 9:**
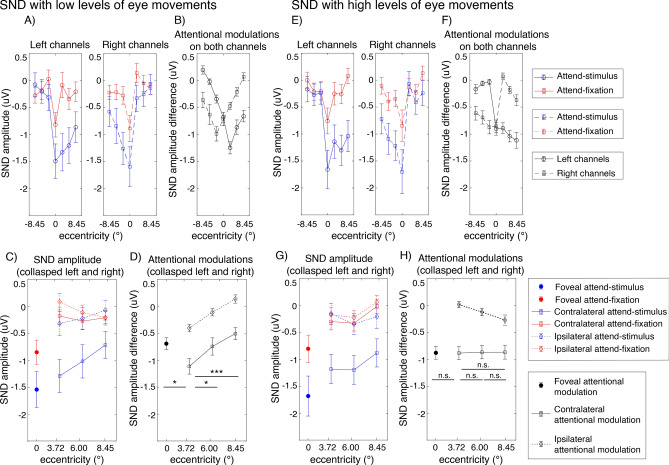
Attentional modulations of the SND amplitudes across eccentricity in trials with low and high levels of eye movements (**A**–**D** and **E**–**H**, respectively). (**A**) The SND amplitudes in the attend-stimulus and attend-fixation conditions measured from the left and right posterior occipital electrodes plotted as a function of eccentricity in trials with low levels of eye movements. (**B**) The difference plot showing the degrees of attention modulations (attend-stimulus minus attend-fixation) from both sets of electrodes plotted in (**A**). (**C**) Same as (**A**) but the data were collapsed across the left and right electrodes. (**D**) Same as (**B**) but the data were collapsed across the left and right electrodes. (**E**–**H**) Similar to (**A**–**D**) but the data were obtained from trials with high levels of eye movements. The error bars in all sub-figures represent the within-subject standard errors of the mean (SEMs). * and *** show significant differences in attentional modulations across eccentric locations with p’s < 0.05 and < 0.001, respectively (two-tailed). *n.s*. non-significant.

In the present study, we found the SND component arising from ~ 300 to 1000 ms after the stimulus onset (Figs. 7, 8). For the foveal stimuli, the SND were distributed centrally at the posterior occipital electrodes and moved more contralaterally as the locations of the stimuli were further away from the fovea. As illustrated in Fig. 9, we observed the higher bilateral SND amplitude (i.e., more negative) elicited by the foveal stimulation compared to the contralateral SND amplitude elicited by the more peripheral locations, resulting in a significant interaction between eccentricity and channel location (left and right posterior occipital electrodes) (F(6,162) = 18.72, p < 0.001). Post-hoc paired *t*-tests showed that the SND amplitudes for the foveal locations were significantly more negative than those in the more peripheral locations (t(27)’s = 2.24–3.36, p’s = 0.002–0.034, two-tailed, passing the Holm–Bonferroni-corrected threshold of 0.05). Additionally, the SND amplitudes in the far peripheral locations were significantly less negative than those in the near peripheral location (t(27) = − 2.81, p = 0.009, two-tailed, passing the Holm–Bonferroni-corrected threshold of 0.0167) but the SND amplitudes in the parafoveal location did not differ from those in the far peripheral locations (t(27)’s = 0.49–1.61, p’s = 0.120–0.631, two-tailed). Together, these data suggested that the amplitude reduction of the SND component towards the peripheral direction occurred in a graded fashion.

Similar to the SSVEP results, we observed significant interactions between attention and eccentricity (F(6,162) = 2.94, p = 0.009) as well as between these two factors and channel location on the SND amplitudes (F(6,162) = 27.88, p < 0.001). Importantly, we found a significant three-way interaction between the level of eye movement, attention, and eccentricity, suggesting that the patterns of attentional modulations of the SND amplitudes across eccentricity were different between trials with the low and high levels of eye movements. Specifically, in trials with low levels of eye movements, we found the heterogeneous pattern of attentional modulations of the SND amplitudes similar to the SSVEP results (Fig. 9B,D). That is there was a significant reduction of attentional modulations of the SND amplitudes at the foveal compared to the parafoveal locations (t(27) = 2.45, p = 0.021, two-tailed) and the degree of attentional modulations of the SND amplitudes also decreased significantly in the peripheral compared to the parafoveal locations (near peripheral vs. parafoveal location: t(27) = − 2.25, p = 0.033; far peripheral vs. parafoveal location: t(27) = − 3.76, p < 0.001, two-tailed). In contrast, we found that the attentional modulations of the SND amplitudes were comparable across all eccentric locations in trials with high levels of eye movements (t(27)’s = 0.01–0.11, p’s = 0.911–0.990, two-tailed) (Fig. 9F,H).

Taken together, the SDN data suggested that the degrees of attentional modulations of the top-down attention signals from the frontoparietal regions also differed across eccentricities when eye movements were carefully controlled and the observed heterogeneity of the pattern of attentional modulations of the SDN signals depend on the level of eye movements.

## Discussion

Despite the fact that foveal and peripheral visions have distinct neural architectures and functions, it has been debated if attention operates similarly or differently across eccentricities. We found that the patterns of attention modulations of both early sensory responses as indexed by the SSVEPs and the fronto-parietal neural activity as indexed by the SND component varied depending on eccentric locations. In trials with a negligible level of eye movement, we found that the degrees of attentional modulations of SSVEPs and SND were maximal at the parafoveal locations. Interestingly, the degrees of attentional modulations of these neural signals reduced at the locations more foveal or more peripheral to the parafoveal locations, resulting in the heterogeneous pattern of the attention effects across different eccentricities. Importantly, the heterogeneity of attentional modulations of the SSVEP and SND data were relatively less robust in trials with higher levels of eye movements. Together, these results suggest that sustained visuospatial attention operates differently across different eccentricities and the effects of covert visuospatial attention on early sensory responses and frontoparietal signals depend on the level of eye movements.

The reduction of the attentional gain modulations of the SSVEP SNR at the locations in our present study were consistent with a previous single-unit recording study that reported the reduced attentional gain of neurons in areas near the fovea of the primary visual cortex in macaque monkeys^14^. The similarity between our SSVEP results in humans and the previous single-unit results in monkeys suggests that the way that attention affects neural gain of early sensory processing near the fovea was highly preserved across the two primate species. One may argue that, unlike single-unit recording data, SSVEP signals provide a rather broad measurement of population-level neural activity, which reflects a mixture of responses across multiple areas (see comprehensive review by Norcia^35^. However, in the current study, we selected a relatively high flicker frequency (i.e., 18.75 Hz) to ensure that the SSVEP responses primarily arose from neural activity within early visual areas (see similar protocols^27,38, 49, 50, 59–61^). With the particular choice of this high flicker frequency, we believe that the attentional modulations of SSVEP data observed in our study primarily reflected attentional modulations of the early visually evoked responses.

The aforementioned single-unit study also discovered that attention reduced spatial summation near the fovea compared to more peripheral locations^14^. In the present study, we could only assess attentional gain modulations of the SSVEP responses and could not directly measure changes in spatial summation. Thus, the comparison between our current data and the spatial summation data in the single-unit study^14^ must be taken with careful consideration. While past studies have attempted to explain the contributions of spatial summation to attentional modulations of neural firing in early visual areas^62–64^, there is no straightforward answer that explains the link between attention-mediated changes in spatial summation and gain modulations. On one hand, the reduced spatial summation could potentially increase the sensitivity of neurons near the fovea leading to an increase in attentional gain modulations of neural responses. However, this will happen only if the size of the stimulus matches the size of the excitatory field, which is not the case in our current task design (i.e., the stimulus size is much bigger than the receptive field of neurons near the fovea). On the other hand, the reduced spatial summation could potentially heighten the spatial resolution of neurons near the fovea without changing the size of the suppressive field. With the fixed stimulus size, the reduced spatial summation would lead to an increase in the suppressive drive, which will then normalize the attentional gain of neuronal outputs. This could result in a relatively lower degree of attentional gain modulations of visuocortical activity at the foveal compared to the more peripheral locations as we observed in our present study. Consistent with this idea, previous studies have suggested that spatial summation of neuronal populations could be explained by divisive normalization of sensory gain modulations, which is thought as a canonical model that explains gain modulations in sensory and attentional systems^50,65–68^.

In contrast to the results reported by the previous monkey study^14^, a recent EEG study has argued that the same gain mechanism underlies the effects of attention on the foveal and peripheral vision in humans^15^. They found the robust attentional modulations of the early visually evoked potential (VEP) were observed when visual stimuli were presented near the fovea^15^. Note that even though they found significant effects of attention on the VEP evoked by the stimuli near the fovea, they did not systematically compare the results across different eccentric locations. Thus, this single demonstration is insufficient for concluding that attention operates via similar gain mechanisms across eccentricities.

The reduction in the attentional modulations of the SSVEP (in the present study) and single-unit data at the fovea could be due to the possibility that the ignored stimuli at the fovea can compete for more attentional resources than the ignored stimuli at more peripheral locations. Specifically, in the attend-fixation condition, as the visual stimulus approached fixation, it became more likely to interfere with the behavioral task at fixation. Due to its closer proximity to the fixation point, the foveal flicker had greater overlap with the target, where subjects were tasked with detecting changes in stimulus contrast, potentially resulting in less suppression compared to the parafoveal flicker. While these alterations in distractor suppression related to eccentricity might explain the decrease in attentional modulations at foveal locations compared to parafoveal ones, they fail to elucidate the decline in attentional modulations at peripheral locations relative to parafoveal ones. Additionally, contrast thresholds and hit rates in the attend-fixation conditions remained constant across eccentricities. Note that the reduced attentional modulations at the fovea observed in our study should not be influenced by the saturation of the SSVEP signals because the stimuli were presented at 50% contrast. Though one might argue that 50% contrast may be still too high, many prior SSVEP studies have shown that SSVEP responses did not saturate at 50% contrast or sometimes they did not saturate at all even at 100% contrast^27,38, 59, 60, 69, 70^. Therefore, response saturation should not be a particular concern for the current task design.

As the eccentric locations of attention and visual stimuli were further away from the parafoveal locations in the peripheral direction, the attentional modulations on the SSVEP and the SND signals also decreased. This is consistent with the behavioral results where contrast sensitivity and behavioral accuracy decreased as a function of eccentricities in the attend-stimulus compared to the attend-fixation conditions in trials with low levels of eye movements. Based on past studies, differences in task difficulty could lead to varying degrees of attentional gain modulations in the early visual cortex^59,71–78^. Driven from this idea, the increase in perceptual difficulty at processing visual stimuli in the periphery could contribute to this reduction in the attentional modulations of the SSVEP and SND signals observed in the present study.

The similar decreases in the attentional modulations in the periphery were observed in the fMRI data measured in several regions within the early visual, ventral, and lateral occipital areas^16^. The drop-off of the attention-related neural signal in the periphery has also been reported by a recent EEG study that measured the N2pc, the contralateral-vs-ipsilateral ERP difference, commonly known to index target selection processes^79^. The differences between our SND and the recent N2pc results were that the SND modulations sustained over a much longer period of time and that the SND data were obtained from the non-target presented trials while the N2pc data were directly related to the targets. Therefore, the observed modulations in our SND data reflected the effects that sustained attention had purely on sensory processing and were not influenced by any target- or response-related processes.

The overall heterogeneity in the pattern of attentional modulations suggests differing spatial resolution requirements for target selection. Consistent with this idea, the size of the checkerboard flicker (~ 1.53° in radius) employed to elicit SSVEP responses in the present study closely matched the receptive field (RF) size of early visual areas (V1–V3) at the parafoveal locations. Since the stimulus size was fixed, it was larger and smaller than the RF sizes at more foveal and peripheral locations, respectively^41,42, 45–47, 80, 81^. Accordingly, the optimal spatial resolution at parafoveal locations may result in the attended flicker producing the greatest attentional modulations of the SSVEP response compared to foveal and peripheral locations, where the spatial resolution for selecting a target of this particular size was either too high or too low, respectively. This interpretation aligns well with prior findings indicating superior texture discrimination in parafoveal and mid-peripheral regions compared to more foveal and peripheral ones^82–85^. However, this should be interpreted with caution because we did not manipulate stimulus size to match different RF sizes across eccentricities. Moreover, it is hard to match stimulus size and the RF size across all of multiple regions within the visual cortex and determine the optimal spatial resolution at each eccentricity, which may also rely on the nature and complexity of behavioral tasks. Also note that in our contrast detection task, we did not observe superior behavioral performance at the parafoveal compared to the more foveal and peripheral locations. Thus, at least in the context of the recent study, the spatial resolution account could not explain the link between the heterogeneity pattern of the attentional modulations of the neural responses and the pattern of the behavioral data. Future research could adapt our experimental design to investigate how visuocortical responses are jointly modulated by eccentricity, spatial resolution, levels of visual processing (e.g., contrast, orientation, texture, and higher processing), and the nature of behavioral tasks (e.g., detection vs. discrimination).

According to prior studies, there are two alternative methods employed to examine attentional modulations of cortical responses across eccentricities^10,16^. On one hand, a visual stimulus of fixed size and spatial frequency could be presented across different eccentricities, and behavioral and neural responses could be compared across eccentric locations^10^. Indeed, past studies have also used a behavioral paradigm with fixed stimulus properties to estimate cortical magnification factor^44^. Alternatively, magnification scaling could be done to minimize the impact of cortical magnification on the pattern of the data^16^. Here, we chose the method of constant stimuli because we wanted to estimate how contrast threshold and SSVEP responses change across eccentricities to ensure that our manipulation of stimulus eccentricity worked in terms of generating different levels of behavioral thresholds and SNR of SSVEP signals across eccentricities. Since we did not correct for cortical magnification, it is reasonable to expect that behavioral thresholds and task difficulty would be relatively higher in more peripheral locations compared to foveal locations, as illustrated in Fig. 1. Past studies suggest that increasing perceptual difficulty may enhance the impact of attention on early sensory responses^59,73–78, 86^. Accordingly, one might anticipate a greater degree of attentional modulation of SSVEP and SND at more peripheral locations based on these findings. However, our observations revealed a contradictory pattern where attentional modulations of SSVEP and SND decreased with eccentricity at the peripheral locations and also when the stimulus shifted from parafoveal to foveal locations. Due to these contradictory findings, the pattern of the results could not be solely explained by the influence of cortical magnification. That said, it is important to note that the heterogeneous pattern of attentional modulations observed across eccentricities could be influenced by the switches in contribution of the parvocellular and magnocellular pathways to responses measured across different eccentricities (see further discussion in the following paragraph).

Functional segregation between central and peripheral vision involves two distinct cellular pathways known as the parvocellular and magnocellular pathways, respectively^87–91^. While it is important to dissociate the roles of these two cellular pathways in mediating attention effects across eccentricities, it is beyond the scope of the current study. Indeed, by design, we chose the black-and-white stimulus flicker of relative high temporal frequency (i.e., 18.75 Hz) but low spatial frequency (~ 1 cpd) to mainly target the magnocellular pathway following prior studies^92–94^. We decided to target the magnocellular pathway for two main reasons. First, non-human primate studies have shown that magnocellular neurons are more evenly distributed across eccentricity compared to parvocellular neurons^90,91^. Second, the magnocellular pathway is thought to be more predominantly involved in top-down control of visual spatial attention, which is the main focus of the current study^87,95–102^.

In addition to spatial and temporal frequencies, stimulus luminance and contrast are other key factors that may cause preferential bias in the parvocellular vs. magnocellular pathways. For example, a prior study has shown that neural responses of the magnocellular pathway in lateral geniculate nucleus (LGN) decreased significantly after the luminance reached only about ~ 7 cd/m^2^, while neural responses in the parvocellular pathway were maintained at the similar level throughout^103^. That said, in this particular study, the stimuli were presented on the black background of 0.2 cd/m^2^, unlike the present study where the background had a much higher luminance value (42.05 cd/m^2^). Indeed, many past studies have also used backgrounds of higher luminance values (40–150 cd/m^2^)^104–108^. Since the background values were relatively higher in these studies, levels of neural responses were examined as a function of Michelson contrast instead of luminance^104–108^. Overall, they found neural responses of the magnocellular pathway peaked at around 50% Michelson contrast. Importantly, these responses either stayed at the similar level or dropped as the contrast values increased above 50%^110–114^. Note that in these studies, neural responses of the parvocellular pathway increased dramatically after the Michelson contrast value reached ~ 50%^104–108^. According to these findings and given our current design, where we selected the stimulus flicker of high temporal and low spatial frequencies, we think the Michelson contrast of 50% was an optimal option to target the magnocellular pathway. That said, while temporal and spatial properties and physical contrast chosen for the present study may produce preferential bias to the magnocellular pathway, it is still a ‘bias’ rather than an ‘absolute’ preference. Therefore, it is crucial to interpret the findings of this study with careful attention to the fact that the central and peripheral visual pathways exhibit distinct variations in photoreceptor densities, distributions, and types. We suggest that future studies should investigate these issues further by systematically manipulating luminance and contrast values as well as temporal and spatial frequencies of visual stimuli.

Finally, in trials with a relatively higher level of eye movements, the overall SNR of the SSVEP signals decreased and the heterogeneous pattern of attentional modulations of the SSVEP signals became less robust. We believe this was due to the possibility that eye movements reduced spatial overlaps between stimulus presentations across different trials resulting in the decrease in synchronous activity indexed by the SSVEP signals. While the significant heterogeneous pattern of attentional modulations was still observed in the SSVEP data, the degrees of attentional modulations of the SND data were comparable across eccentricities in trials with the higher level of eye movements. These findings suggest that the effects of covert visuospatial attention on SSVEPs are less susceptible to eye movement artifacts than those of ERP measurements like the SND component. That said, distinct patterns of attentional modulations of the SSVEP and SND signals in trials with varying degrees of eye movements suggest that these neural signals and EOG could be integrated to develop a hybrid BCI system that can track covert visuospatial attention with a precise eye movement control^109–114^. In the current study, we did not collect eye movement data using an eye tracker, which could have provided more precise estimates of eye positions. Therefore, we could not examine how the directions of small eye movements might influence the EEG data. That said, we believe the spatial resolution of the EOG data sufficiently served the purpose of the current study, as our sole objective was to ensure subjects maintained fixation and minimized excessive eye movements. It is important to note that even small eye movements detected by EOG could substantially influence the patterns of the EEG results. Thus, this underscores the potential confounding effects that small eye movements may contribute to discrepancies observed in previous research findings.

In conclusion, our study provided strong evidence suggesting that attention results in the different patterns of visuocortical response modulations across eccentricities, standing in contrast to a recent proposal by Frey^15^ and others. These results suggest that sustained visuospatial attention operates differently across different eccentricities, providing new insights how attention augments sensory representations across the entire visual field.

## Materials and methods

### Subjects

We recruited 40 neurologically healthy human adults who had normal or corrected-to-normal vision from the community surrounding Vanderbilt University, Tennessee to participate in the experiment. Their ages ranged from 18 to 39 years old. Among these subjects, 31 subjects completed the experimental protocol, which included 2 days of EEG sessions. Prior to their participation, they provided written informed consent as required by the local Institutional Review Board at Vanderbilt University and were compensated at a rate of 10 USD per hour of participation. The data from 2 subjects were excluded from the final analysis because we could not equate task difficulty across eccentricities in these individuals. Specifically, one of them produced spurious false alarms while detecting the target at the furthest peripheral locations (p(FA) = 0.30 and 0.28 for left and right) compared to the foveal locations (p(FA) = 0.04), another subject failed to detect the target at the rightmost peripheral locations (p(hit) = 0.51) compared to the foveal locations (p(hit) = 0.74). Moreover, we excluded the data from another subject because their eye scores based on the EOG data were above 16 uV, corresponding to the deviation of eye movement more than 10° visual angel from the central fixation. These exclusion criteria left the data from 28 subjects in the final analysis and results reported here (15 female, 2 left-handed, mean age = 23.36 ± 4.139 years old).

### Consent statement

The research protocol was approved by the ethics committee of the institutional review board (IRB) at Vanderbilt University and conducted in accordance with the Declaration of Helsinki.

### Stimuli and behavioral tasks

Stimuli were presented on a Macintosh desktop running MATLAB 7.10.0 (R2010A) (Mathworks Inc., Natick, MA) and the Psychophysics Toolbox: version 3.0.8^115,116^. The subjects were seated 80 cm from the CRT monitor with a gray background of 42.05 cd/m^2^ (refresh rate = 75 Hz, 800 × 600 resolution). The experiment was performed in a dark, sound-attenuated, and electromagnetically shielded room (ETS-Lindgren, Cedar Park, TX, USA).

While EEG signals were being recorded, the participants performed two attention tasks, consisting of attend-fixation and attend-stimulus tasks (Fig. 1A). In the attend-fixation task, the subjects fixated at the small 100% contrast checkered circle (diameter = 0.3° visual angle), while ignoring the larger 50%-contrast checkerboard stimulus (spatial frequency = 1.03 cpd; diameter = 3.06° visual angle) that flickered at 18.75 Hz at one of 22 possible spatial positions on each trial along an elongated hexagonal grid. Note here that the fixation circle did not flicker and it stayed on the screen throughout the entire experiment. We chose a relatively high frequency (18.75 Hz) with a relatively low spatial frequency (~ 1 cycle per degree) to target the magnocellular pathway, since it known to be sensitive to visual stimuli with high temporal frequencies (more than 5 Hz) but low spatial frequencies^92–94^ (see rationales in the “Discussion” section).

For the 100%-contrast small stimulus at the fixation, we used the checkerboard, of which dark and light areas had the luminance values of 0.1 and 84.0 cd/m^2^, respectively. For a larger 50%-contrast flickering stimulus, the dark and light areas had the luminance values of 21.08 and 63.03 cd/m^2^, respectively. Here, the contrast values of the stimuli were determined using the Michelson contrast equation: C_Michelson_ = (L_max_ − L_min_)/(L_max_ + L_min_), where L_max_ and L_min_ are the maximum and minimum luminance.

The hexagonal grid consisted of three rows (Fig. 1B). The middle row contained 8 equally spaced spatial positions extending to the left and right of the central fixation along the horizontal meridian (1.29°, 3.87°, 6.45°, 9.03° visual angle to the left and the right of the fixation). The upper row contained 7 equally spaced spatial positions aligned in parallel with the middle row 2.58° above the horizontal meridian. This yielded one stimulus that was placed at 2.58° visual angle above the central fixation and the other 6 stimuli placed at − 8.16°, − 5.77°, − 3.65°, 3.65°, 5.77°, 8.16° visual angle from the central fixation in the upper left (negative values) and upper right quadrants (positive values). The bottom row also consisted of 7 equally spaced spatial positions aligned in parallel with the upper row with 2.58° visual angle below the horizontal meridian. This yielded one stimulus that was placed at 2.58° visual angle below the central fixation and the other 6 stimuli placed at − 8.16°, − 5.77°, − 3.65°, 3.65°, 5.77°, 8.16° visual angle from the central fixation in the lower left (negative values) and upper right quadrants (positive values).

Each block contained 220 trials where the large 50%-contrast flickering checkerboard stimuli could appear at one of these 22 locations (10 repeats of each location) for 1000 ms, followed by the intertrial interval (ITI) pseudo-randomly drawn from the uniform distribution of 500–1000 ms. We used 50% stimulus contrast here to avoid response saturation, and past studies have shown robust attentional modulations of behavioral responses and neural activity in the early visual areas when the spatial scopes of attention were relatively broader like in the current design^50,66, 67, 117–119^.

In the attend-fixation task, subjects were instructed to fixate at the small 100%-contrast checkerboard at the central fixation for the whole block to detect a contrast decrement at the fixation that occurred in 20% of the trials (2 trials for each of the 22 locations of the large flickering 50%-contrast checkerboard stimuli). The contrast decrement at the fixation could occur from 150–650 ms after the onset of the large 50%-contrast flickering checkerboard stimulus, and the constant contrast decrement at the fixation stayed on for 300 ms before returning to the baseline level of 100% contrast. The trial sequence was shuffled so that subjects could not predict trial types and the locations of the to-be-ignored stimuli. We adjusted the levels of the contrast decrement at the fixation block-by-block to maintain hit rates of ~ 0.7. We did this separately for when the large checkerboard stimuli appeared at the fovea (collapsed between 1.29° to the left and right of the fixation and 2.58° below and above the fixation, marked by yellow circles in Fig. 1B; mean eccentricity = 0°), the left/right parafoveal locations (mean eccentricity of ± 3.72° marked by the green circles), the left/right near peripheral locations (mean eccentricity of ± 6.00° marked by cyan circles) and the left/right far peripheral locations (mean eccentricity of ± 8.45° marked by the blue circles).

The attend-stimulus task was identical to the attention-fixation task except that the contrast of the small 100%-contrast checkerboard at the central fixation would not change. However, on 20% of the trials (2 trials for each of the 22 locations), the large 50%-contrast flickering checkerboards that appeared at the foveal, parafoveal and peripheral locations would dim slightly for 300 ms anytime from 150–650 s after the stimulus onset. The subjects were instructed to maintain fixation while covertly attending to the large flickering 50%-contrast stimuli at the parafoveal and peripheral locations to detect contrast decrements. The levels of contrast decrements were adjusted block-by-block to maintain hit rates at about 0.7 for all eccentricities (collapsed into 7 locations; see Fig. 1C). For both attend-fixation and attend-stimulus tasks, the subjects had to press a button on the keypad as fast and correctly as possible when they saw the contrast increment. Note that in the attend-stimulus task, there were multiple eccentric locations where we measured contrast discrimination thresholds from, thus it was challenging to use attention cues to guide attention on a trial-by-trial basis. Instead, we manipulated attention by giving subjects different instructions on a block-by-block basis. Each subject completed 8–10 blocks of each task across the 2 days of the experiments (1760–2200 trials in total for each task for each subject). We switched between the attend-fixation and attend-stimulus tasks every block, and block sequences (e.g., attend-fixation first or attend-stimulus first) were counterbalanced across subjects. Each block lasted about 6.5 min, and the entire experiment lasted about 2–2.5 h including EEG preparation on each day.

### Behavioral analysis

We first computed the algebraic means of the hit and false alarm rates for all attention conditions and 7 sets of the eccentricity levels: 1 foveal, 2 parafoveal (left/right), 2 near peripheral (left/right) and 2 far peripheral locations (left/right) (see Fig. 1D,F).

First, we use one-way repeated-measures ANOVAs with a within-subject factor of stimulus eccentricities (7 levels: S − 8.45°, − 6°, − 3.72°, 0°, + 3.72°, + 6° and + 8.45° visual angle, where 0° is the fovea and – and + values indicate the averaged eccentricity relative to the left and right of the fovea) on the contrast thresholds separately for the attend-fixation and attend-stimulus conditions. Note that we did not examine the main effect of attention or the interaction between attention and eccentricity on the contrast thresholds because the data between the attend-fixation and attend-stimulus tasks were measured from different sets of visual stimuli that had different sizes and base-line contrast levels (i.e., the small fixation of 100% contrast vs. the much larger checkerboard stimulus of 50% contrast). Thus, the data between the two tasks were expected to be different based on the physical properties of the stimuli and the fixation, and the data should not be compared across attention conditions.

To test if the task difficulty was equated across the different attention conditions and eccentricities, we use 3-way repeated-measures ANOVAs with the within-subject factors of attention (2 levels: attend-fixation vs. attend-stimulus), eccentricity (7 levels: − 8.45°, − 6°, − 3.72°, 0°, + 3.72°, + 6° and + 8.45° visual angle), and the level of eye movements (2 levels: low vs. high) measured via EOG to test the main effects of and the interaction between these two factors on hit and false alarm rates. We then used 2-way repeated-measures ANOVAs to examine the main effects of attention and eccentricity on hit and false alarm rates separately in trials with low and high levels of eye movements.

### EEG recording

We recorded EEG data from the 10–20 sites, including Fz, Cz, Pz, F3, F4, C3, C4, P3, P4, PO3, PO4, O1, O2, T3, T4, T5, and T6, and a pair of custom sites OL and OR, which were halfway between O1 and T5 and halfway between O2 and T6, respectively. These EEG data were referenced online to the right mastoid. We monitored blinks and vertical eye movements using an electrode placed below the right eye and tracked horizontal eye movements via a pair of external electrodes affixed ~ 1 cm lateral to the outer canthi of the left and right eyes. The impedance of each electrode was kept below 3 k-Ohm. The EEG data were amplified with a gain of 20,000 using an SA Instrumentation amplifier with a bandpass filter of 0.01–100 Hz at the sampling rate of 250 Hz.

### EEG preprocessing

We preprocessed the EEG data using custom MATLAB scripts and EEGLab11.0.3.1b^120^. First, we re-referenced the EEG data offline to the average of the left and right mastoid electrodes. Next, we filtered the data using 0.1-Hz high-pass and 55-Hz low-pass Butterworth filters (3rd order). Next, we segmented the continuous EEG data into epochs extending from 2 s before to 2 s after stimulus onset, and the baseline activity averaged from 0–0.2 s before stimulus onset was subtracted from the EEG data. We then performed independent component analysis (ICA) to remove prominent eye blinks^121^ and used threshold rejection and visual inspection to reject trials containing saccades, muscle activity, drifts, and other artifacts. This artifact rejection protocol resulted in the removal of 12.28 ± 4.89% SD of trials across the 28 subjects. The threshold rejection method was used here to disregard epochs contaminated by prominent saccades that could be observed on a trial-by-trial basis. The EOG rejection thresholds for individual subjects were adjusted so that the averaged eye score for each attention condition and each eccentricity was below 1.6 μV, which was about 1° visual angle^122^. As a further step to minimize potential confounds from residue eye movements on the EEG data, we sorted the data into trials with low and high levels of eye movements using the median split method. Specifically, we obtained the eye score from each trial by computing the absolute value of the difference between the maximum and minimum values of EOG activity and then divided the EEG (and behavioral) data in each attention condition and each eccentricity into trials with low and high levels of eye movements.

### EEG analysis

The artifact-corrected EEG epochs were sorted into 28 bins: 2 attention conditions (attend-stimulus and attend-fixation) × 7 eccentricities (+ 8.45°, − 6°, − 3.72°, 0°, + 3.72°, + 6°, and + 8.45° visual angle, where 0° is the fovea and – and + signs indicate the averaged eccentricity to the left and right of the fovea) × 2 levels of eye movements (low and high). To minimize confounds from target- and response-related brain processes, we included only correctly rejected non-target trials (i.e., excluding all target trials and non-target trials with false alarms). These data were used for examining the effects of attention, eccentricity, and eye movement as well as their interaction on two neural markers of visual information processing, consisting of the steady-state visually evoked potential (SSVEP) and the sustained negative deflection (SND).

First, we examined the attentional modulations of the SSVEP signals, which were the phase-locked visually evoked EEG responses that oscillated at the same frequency as the frequency of the flickering visual stimulus thought to be generated from the early visual areas^34,35, 54^. Many past studies have implicated that the attention-induced increases in the SSVEP power/amplitude reflect the attentional gain amplification of the population-level early sensory responses^27,38, 48, 49, 51–57, 69^.

To obtain SSVEPs, we first averaged the EEG data across trials to obtain the event-related potentials (ERPs) for individual bins. Then, we filtered the data with a Gaussian wavelet function with a 0.1 fractional bandwidth to obtain frequency-domain coefficients from 6.75 to 30.75 Hz in 1 Hz steps. SSVEPs evoked by the individual stimulus flicker frequency of 18.75 Hz were then obtained by computing the power of the coefficients at the center of flicker frequency. The signal-to-noise ratio (SNR) of the SSVEPs were then calculated by dividing the power at the flicker frequency of 18.75 Hz by the averaged power of surrounding frequencies including 14.75, 15.75, 21.75, and 22.75 Hz. We then collapsed the SSVEP data into 7 sets of stimulus locations following the behavioral analysis (− 8.45°, − 6°, − 3.72°, 0°, + 3.72°, + 6° and + 8.45° visual angle). We focused the analyses on the left and right posterior occipital electrodes where the signals peaked (i.e., PO3, O1 and OL for the left channels and PO4, O2, and OR for the right channels). Since past studies have found that the effect of sustained attention of SSVEPs started at ~ 300 post-stimulus, we averaged the data from 300–1000 ms^51,58^. We then plotted the SSVEP SNR from the left and right posterior occipital electrodes across 7 sets of eccentric locations separately for individual attention conditions and trials with low and high levels of eye movements (Fig. 5). We then used a 4-way repeated-measures ANOVAs to test the main effects of attention (2 levels: attend-fixation and attend-stimulus), eccentricity (7 levels: − 8.45°, − 6°, − 3.72°, 0°, + 3.72°, + 6° and + 8.45° visual angle), channel location (2 levels: left and right posterior occipital electrodes), and eye movement (2 levels: low and high) as well as their interactions on the SNR of the SSVEP signals.

Since the SSVEP signals were evenly distributed across the left and right channels for the foveal stimulation, we collapsed the data across the two hemispheres. In contrast to the foveal stimulation, the parafoveal and peripheral stimulations produced the SSVEP signals that peaked over the contralateral electrodes. Thus, for all parafoveal and peripheral locations, we analyzed the data from the contralateral and ipsilateral electrodes, separately. Since the data from the left and right stimulations were qualitative similar, we collapsed the contralateral and ipsilateral responses related to the peripheral stimulation to the left and the right of the central fixation, resulting in 3 different sets of eccentricities for the parafoveal and peripheral locations (mean eccentricities = 3.72°, 6.00°, and 8.45° visual angle). Due to this data collapsing step, the contralateral and ipsilateral SSVEP signals related to the parafoveal and peripheral stimuli were obtained from both left and right channels, identical to the sets of electrodes where we obtained the foveal responses. This allowed the same-electrode comparisons between the bilateral SSVEP signals elicited by the foveal stimulation and the contralateral SSVEP signals elicited by the parafoveal and peripheral stimulations which we examined statistically using paired *t*-tests.

Since the overall SSVEP responses could be canceled out by combining responses across the upper and lower VFs, we performed an auxiliary analysis, where we examine SSVEP responses separately for visual stimuli in the upper and lower VFs (in trials with low levels of eye movements). First, we used a three-way repeated-measures ANOVA to test the main effects of and the interactions between attention, eccentricity, and vertical positions (upper vs. lower VFs) on the SSVEP SNR. Next, we performed other sets of two-way repeated-measures ANOVAs to test the interaction between attention and eccentricity separately for the data obtained from the upper and lower VFs.

Note that we focused our analysis on SSVEPs instead of other early sensory responses like the P1 component, which typically occur ~ 70–100 ms after stimulus onset because a previous study has argued that visual stimulation with a sharp onset could automatically capture attention, and this could potentially mitigate the effects of endogenous attention on the P1 component^15^. Instead, the SSVEP signals were continuous visually evoked responses recorded over 1000 ms throughout the entire stimulus duration and should not be of this particular concern.

In addition to SSVEPs, we examined the effects of attention and eccentricity on the sustained negative deflection (SND). The SND component is a negative-going ERP that has been shown to track the top-down biasing signals from the fronto-parietal cortex onto the occipital cortex^27,36–39^. The mean amplitudes of the SND component for individual attention conditions and stimulus locations were computed by averaging the stimulus-locked ERPs from 300–1000 ms after stimulus onset across the same sets of left and right posterior occipital electrodes. We then used a 4-way repeated-measures ANOVAs to test the main effects of attention (2 levels: attend-fixation and attend-stimulus), eccentricity (7 levels: − 8.45°, − 6°, − 3.72°, 0°, + 3.72°, + 6° and + 8.45° visual angle), channel location (2 levels: left and right posterior occipital electrodes), and eye movement (2 levels: low and high) as well as their interactions on the SND amplitudes. Next, these SND amplitudes were then subjected to the same data collapsing steps and statistically analyses as the SSVEP data. Since we did not have attention cues in our design, we did not focus our analysis on other ERP components such as early attention directing negativity (EDAN), anterior attention directing negativity (ADAN), late directing attention positivity (LDAP), which have been associated with cue-related processes and preparatory attention^123–128^.

## Data Availability

The data and source codes are available on the Open Data Framework (https://osf.io/wphnj/).
